# *STAT3* mutation impacts biological and clinical features of T-LGL leukemia

**DOI:** 10.18632/oncotarget.18711

**Published:** 2017-06-27

**Authors:** Antonella Teramo, Gregorio Barilà, Giulia Calabretto, Chiara Ercolin, Thierry Lamy, Aline Moignet, Mikael Roussel, Cédric Pastoret, Matteo Leoncin, Cristina Gattazzo, Anna Cabrelle, Elisa Boscaro, Sara Teolato, Elisa Pagnin, Tamara Berno, Elena De March, Monica Facco, Francesco Piazza, Livio Trentin, Gianpietro Semenzato, Renato Zambello

**Affiliations:** ^1^ Padua University School of Medicine, Department of Medicine, Hematology and Clinical Immunology Branch, Padua, Italy; ^2^ Venetian Institute of Molecular Medicine (VIMM), Padua, Italy; ^3^ Department of Clinical Hematology, University Hospital of Rennes, Rennes, France; ^4^ Biology Department, University Hospital of Rennes, Rennes, France

**Keywords:** large granular lymphocyte leukemia, STAT3 mutation, immunophenotype, neutropenia, fas ligand

## Abstract

*STAT3* mutations have been described in 30-40% of T-large granular lymphocyte (T-LGL) leukemia patients, leading to STAT3 pathway activation. Considering the heterogeneity of the disease and the several immunophenotypes that LGL clone may express, the aim of this work was to evaluate whether *STAT3* mutations might be associated with a distinctive LGL immunophenotype and/or might be indicative for specific clinical features.

Our series of cases included a pilot cohort of 101 T-LGL leukemia patients (68 CD8+/CD4- and 33 CD4+/CD8±) from Padua Hematology Unit (Italy) and a validation cohort of additional 20 patients from Rennes Hematology Unit (France).

Our results indicate that i) CD8+ T-LGL leukemia patients with CD16+/CD56- immunophenotype identify a subset of patients characterized by the presence of *STAT3* mutations and neutropenia, ii) CD4+/CD8± T-LGL leukemia are devoid of *STAT3* mutations but characterized by *STAT5b* mutations, and iii) a correlation exists between STAT3 activation and presence of Fas ligand, this molecule resulting highly expressed in CD8+/CD16+/CD56- patients. Experiments with stimulation and inhibition of STAT3 phosphorylation confirmed this relationship. In conclusion, our data show that T-LGL leukemia with specific molecular and phenotypic patterns is associated with discrete clinical features contributing to get insights into molecular bases accounting for the development of Fas ligand-mediated neutropenia.

## INTRODUCTION

T-cell large granular lymphocyte (T-LGL) leukemia is a rare chronic lymphoproliferative disorder characterized by the clonal expansion of CD3+ Large Granular Lymphocytes (LGL) [1–3]. T-LGLs typically exhibit a post-thymic mature effector memory phenotype (CD3+/CD8+/CD57+/CD45RA+/ CD62L-) and variable expression of CD16, CD56 and NK receptors, namely Killer Immunoglobulin-like Receptor (KIR) and CD94/NKG2, indicating that these cells represent late stage fully differentiated cytotoxic T-lymphocytes [4, 5]. In addition to the most common CD8+ T-LGL leukemia, less frequent LGL proliferations with CD4+/CD8^-/+dim^ phenotype (CD4+ T-LGL leukemia) exist, which are characterized by a typical Vβ13.1 expression, frequent association with secondary neoplasia and a pathogenetic relationship with CMV infection [6].

The etiology of T-LGL leukemia is still unknown. Several reports support the hypothesis that chronic antigenic stimulation induces monoclonal LGL proliferation persisting through defective activation induced cell death (AICD). Several pathways are constitutively activated in T-LGL leukemia leading to resistance to apoptosis, such as MAPK/ERK, PI3K/AKT, NF-κB pathways, sphingolipid rheostat and JAK/STAT pathway [7]. Epling–Burnette et al [8] first demonstrated constitutive STAT3 activation with Mcl1 upregulation in T-LGL leukemia. We previously identified both extrinsic (IL-6 trans-signaling) and intrinsic mechanisms (SOCS3 downregulation) contributing to constitutive STAT3 tyrosine phosphorylation [9]. Moreover, somatic *STAT3* and *STAT5b* mutations determining constitutive activation have been reported, the former detected in a proportion of approximately 40% of patients [10, 11] and the latter being associated to aggressive LGL disorders [12] and, as recently reported, to CD4+ T-LGL leukemia patients (6 out of 11 cases) [13]. Some authors reported that *STAT3* genetic lesions are associated with neutropenia [10, 14], although this correlation has not yet been specifically evaluated also in consideration that the pathogenesis of neutropenia is likely to be multifactorial, comprising both humoral and cytotoxic mechanisms [15]. Since normal neutrophil survival is partly regulated by the Fas-Fas ligand apoptotic system, it is suggested that LGL leukemia neutropenia might be mediated by deregulated expression of Fas ligand. Consistently, high levels of circulating Fas ligand have been detected in T-LGL leukemia serum, likely triggering neutrophil apoptosis through the production of secreted Fas ligand [16].

Taking into account the heterogeneity of the disease and the several immunophenotypes that may characterize LGL clone, the aim of this work was to evaluate whether *STAT3* mutations might be associated with a distinctive LGL immunophenotype and/or indicative for symptomatic disease in an initial cohort of 101 patients affected by T-LGL leukemia. Our results demonstrate that, in CD8+ T-LGL leukemia, the CD16+/CD56- immunophenotype is associated with *STAT3* mutations, identifying a more symptomatic and treatment requiring disease. The predictive value of CD8+/CD16+/CD56- immunophenotype to identify *STAT3* mutated and neutropenic patients was also confirmed in a validation cohort of 20 patients from Rennes University (France). The evidence that CD8+/CD16+/CD56- patients were characterized by higher level of Fas ligand, as a consequence of their higher STAT3 phosphorylation, offers a mechanistic explanation for the correlation between STAT3 activation and neutropenia.

## RESULTS

### *STAT3* mutations

**Figure 1:**
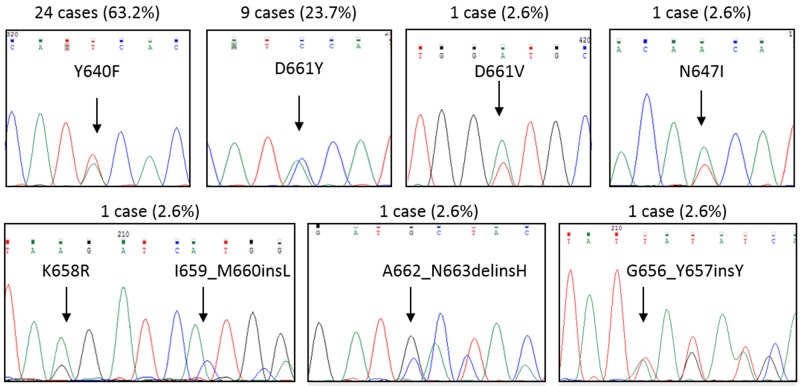
Representative Sanger sequences for each *STAT3* mutation found By Sanger sequencing, *STAT3* mutations were observed in 36 out of 101 T-LGL leukemia patients. Two more cases were found by ARMS-PCR. Upper each graph the cases and their incidence (%) among *STAT3* mutated patients (*n =* 38) are indicated. Y640F and D661Y accounted for the most frequent mutations found.

In the pilot cohort, we observed 38 patients out of 101 analyzed (37.6%) carrying *STAT3* mutations, 36 patients by Sanger sequencing and 2 more cases by ARMS-PCR (amplification refractory mutation system, an assay revealing Y640F and D661Y undetectable by Sanger sequencing if present in less than 25% of cells). All samples were evaluated at diagnosis. *STAT3* mutations were always found in leukemic LGLs and not in the remaining non-leukemic peripheral blood mononuclear cells (PBMCs) (data not shown). In two cases bone marrow cells were available and the same mutation was identified in both peripheral blood and bone marrow. The distribution of mutations was as follows: 24 cases presented Y640F (63.2%), 9 cases D661Y (23.7%), one case D661V (2.6%), one case N647I (2.6%), and 3 cases presented mutations not yet described in T-LGL leukemia. These latter were the following: one case with a point mutation, K658R, together with an in-frame insertion, I659_M660insL (2.6%), a second case with an in-frame deletion/insertion, A662_N663delinsH (2.6%), and a third case with an in-frame insertion, G656_Y657insY (Figure 1). Surprisingly, a significant correlation was demonstrated between the presence of *STAT3* mutations and female gender (χ^2^ = 3.91, *P* < 0.05; Table 1).

**Table 1:** Evaluation of *STAT3* mutations incidence in T-LGL leukemia patients according to clinical characteristics

Variable	T-LGL leukemia patients *n* = 101	Patients with *STAT3* mutation *n* = 38	Patients without *STAT3* mutation *n* = 63	*P*	*STAT3* mutations
**Gender**				0.048	
Males	**50 (50%)**	14 (37%)	36 (57%)		8 Y640F, 2 D661Y, 1 D661V, 1 N647I, 1 G656_Y657insY, 1 K658R and I659_M660insL
Females	**51 (50%)**	24 (63%)	27 (43%)		16 Y640F, 7 D661Y, 1 A662_N663delinsH
**Neutropenia (ANC < 1,500)**	**39 (39%)**	34 (89%)	5 (8%)	0.0001	21 Y640F, 8 D661Y, 1 D661V, 1 N647I, 1 G656_Y657insY, 1 K658R and I659_M660insL, 1 A662_N663delinsH
**Severe neutropenia (ANC < 500)**	**17 (17%)**	17 (45%)	0	0.0001	10 Y640F, 4 D661Y, 1 N647I, 1 K658R and I659_M660insL, 1 A662_N663delinsH
**Rheumatoid arthritis**	**3 (3%)**	3 (8%)	0	0.024	2 Y640F, 1 D661Y
**Other autoimmune diseases**	**23 (23%)**	16 (42%)	7 (11%)	0.0001	11 Y640F, 4 D661Y, 1 K658R and I659_M660insL
**Associated neoplasia**	**28 (28%)**	8 (21%)	20 (32%)	0.245	5 Y640F, 2 D661Y, 1 K658R and I659_M660insL
**Treatment**	**13 (13%)**	12 (32%)	1 (2%)	0.0001	8 Y640F, 3 D661Y, 1 G656_Y657insY

ANC, absolute neutrophil count.

### Clinical characteristics in T-LGL leukemia patients

Among the 101 patients (50 males, 51 females) of the pilot cohort, neutropenia (absolute neutrophil count, ANC < 1,500 mm^3^) was documented in 39 patients (38.6%), with 17 (16.8%) presenting severe neutropenia (ANC < 500 mm^3^) (Table 1). Thirty-four out of 39 neutropenic patients were *STAT3* mutated (87.2%), all the patients characterized by severe neutropenia being included among the group of mutated cases. Only 5 patients were neutropenic and did not display *STAT3* mutations. This correlation between the presence of *STAT3* mutations and neutropenia was demonstrated highly statistically significant (χ^2^ = 66.5, *P* < 0.0001; Table 1 ). No correlation between neutrophils’ count and the kind of *STAT3* mutation or percentage of mutated clone was found. In fact, a patient mutated only by ARMS-PCR had severe neutropenia, vice-versa a patient homozygous for mutation has never experimented severe neutropenia. Similarly, no correlation between neutrophils’ count and LGL percentage or LGL absolute number were observed (data not shown).

Rheumatoid Arthritis (RA) was present in three patients (3%), all mutated and neutropenic. The frequency of RA was lower than previously reported in the literature [17, 18], this can be explained because in our cohort only symptomatic patients who required specific treatment were considered affected by RA; a Reumathoid factor or anti-Cytrulline antibody positivity was not considered sufficient to make diagnosis of RA. Anyway, the association between RA and *STAT3* mutation described in previous study [10] was confirmed. The association with different autoimmune disorders (i.e., autoimmune hemolytic anemia, immune thrombocytopenia, Hashimoto Thyroiditis, Systemic Erythematosus Lupus and Vitiligo) was observed in 23 patients (22.8%), mostly mutated patients (16/23, 69.6%; χ^2^ = 12.9, *P* < 0.0001). Differently, concomitant second neoplasia (benign or malignant), affecting 28 out of 101 patients (27.7%), was not statistically associated with wild type or mutated patients (χ^2^ = 1.35, *P* = 0.245; Table 1).

According to our policy to treat patients when symptomatic for neutropenia and not according to neutrophils number, at the time of the study only 13 patients required treatment, 12 out of 13 were *STAT3* mutated (92.3%; χ^2^ = 19.0, *P* < 0.0001; Table 1). The first perspective trial evaluating the efficacy of immunosuppressive therapy in LGL leukemia evidenced the relationship between the presence of Y640F *STAT3* mutation and response to MTX [19]. In our cohort, 6 patients were Y640F mutated and received MTX therapy obtaining an overall response rate of 67%, with one complete response. Even if in a low number of cases, this result supports the previous data described by Loughran et al [19].

### Immunophenotypic characterization of patients T-LGLs

By flow analysis, we observed that 68 out of 101 patients (67.3%) were characterized by classical CD3+/CD8+/CD4- expression (CD8+ T-LGL leukemia), while the remaining 33 patients (32.7%) were distributed among CD3+/CD4+/CD8+^dim^ (*n* = 23, 22.8%) and CD3+/CD4+/CD8- (*n* = 10, 9.9%) phenotypes. From now on we report these two latter subsets together under the definition of CD4+ T-LGL leukemia. According to the expression of the NK cell markers CD16, CD56 and CD57, several possible immunophenotype combinations were demonstrated in both CD8+ and CD4+ T-LGL leukemia. More specifically, according to positivity and negativity of the three markers listed above, CD8+ T-LGL leukemia displayed five different immunophenotypes, while CD4+ T-LGL leukemia exhibited only two dominant phenotypes (data not shown).

All *STAT3* mutated (*n* = 38) and almost all neutropenic (38 out of 39) patients belonged to CD8+ T-LGL leukemia (*n* = 68), while among CD4+ T-LGL leukemia (*n* = 33) only one neutropenic patient (1 out of 33, 3%) was found, this last characterized by mild neutropenia (ANC: 1,470 mm^3^) over 9 years mean follow up.

**Figure 2:**
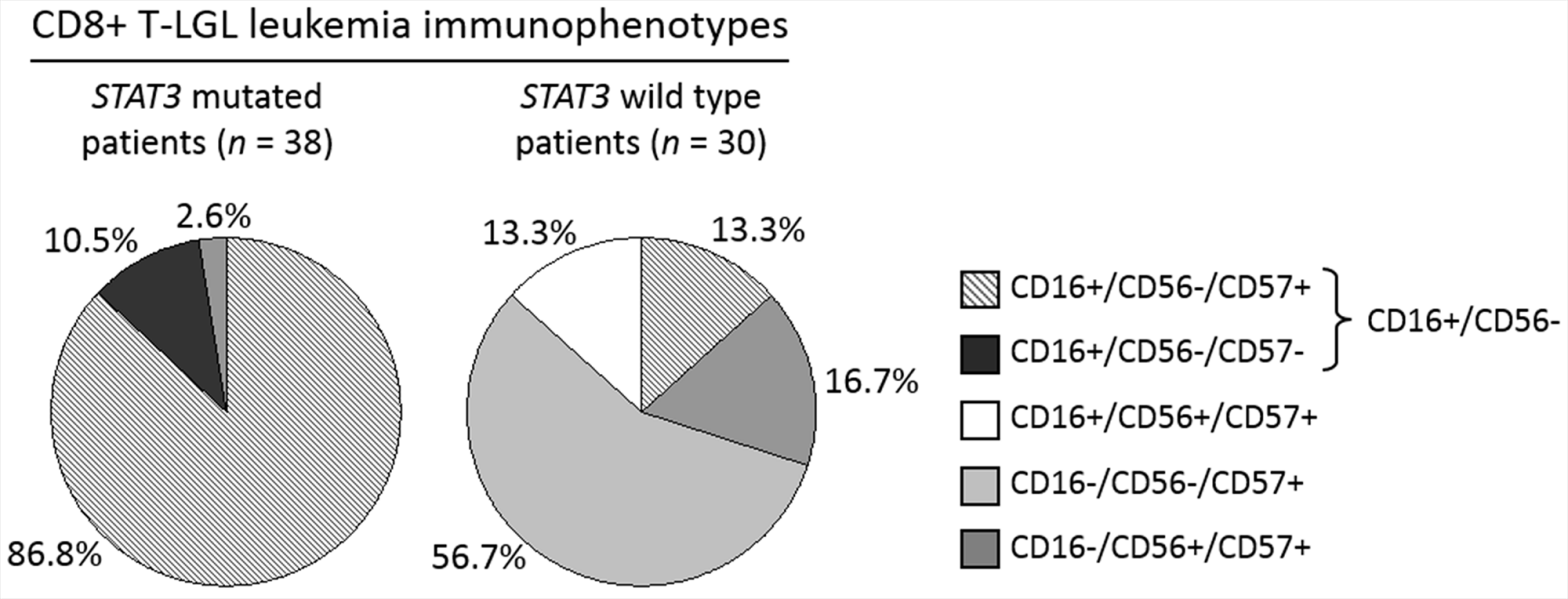
Immunophenotype distribution analysis evaluated in *STAT3* mutated patients as compared with *STAT3* wild type patients within CD8+ T-LGL leukemia The graphs represent the incidence (%) of each LGL immunophenotype in the group of patients with *STAT3* mutations (pie chart on the left) and in the group wild type for *STAT3* gene (pie chart on the right). Mutated patients mostly belong to CD16+/CD56- phenotype (37/41, 90.2%), representing only 13.3% (4/30) of wild type patients’ group.

**Figure 3:**
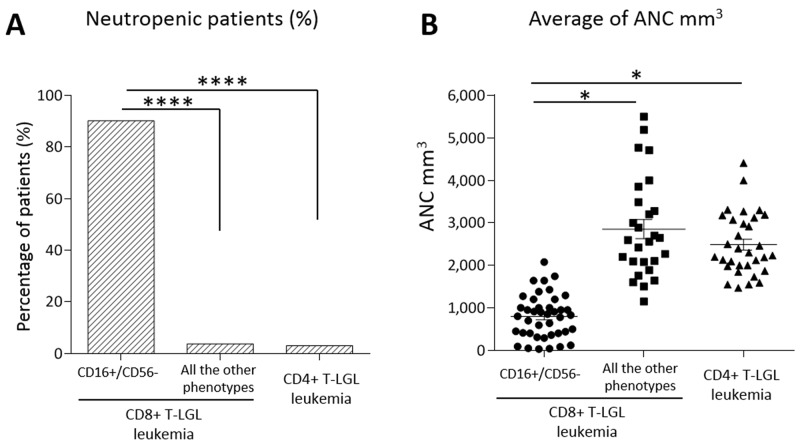
Neutropenia evaluation in the patients subdivided according to their immunophenotypes **(A)** Histogram of the percentages of patients with neutropenia (ANC < 1,500 mm^3^). Neutropenia incidence resulted 90.2% in CD16+/CD56- subset (*n* = 41), 3.7% in the other immunophenotypic subsets of CD8+ T-LGL leukemia (*n* = 27) and 3% in CD4+ T-LGL leukemia (*n* = 33), the difference is highly statistically significant (****P < 0.0001, χ^2^ = 49.5 and χ^2^ = 55.7, respectively, using χ^2^ test). **(B)** Dot plot indicating ANC level of each patient. The mean of ANC ± SEM in CD16+/CD56- subset (795.46 ± 80.03 mm^3^) is lower than in the other immunophenotypic subsets of CD8+ T-LGL leukemia (2,855.11 ± 224.54 mm^3^) and in CD4+ T-LGL leukemia (2,635.76 ± 193.81 mm^3^; *P < 0.05, using one-way Anova and Tukey’s multiple comparison test).

Interestingly, all the *STAT3* mutated samples were mostly characterized by CD16+/CD56-/CD57+ (33 out of 38, 86.8%), followed by CD16+/CD56-/CD57- phenotypes (4 out of 38, 10.5%; Figure 2, left pie chart). At variance, in CD8+ T-LGL leukemia wild type group (*n* = 30), these two immunophenotypes were barely detected (4 out of 30, 13.3%) or absent, respectively (Figure 2, right pie chart). Only one patient with mutated *STAT3* had a different markers expression, being CD16-/CD56-/CD57+ (1 out of 38, 2.6%; Figure 2, left pie chart). In conclusion, the two main immunophenotypes of *STAT3* mutated group were characterized by CD16 positivity and CD56 negativity, defining an immunophenotype signature CD16+/CD56- (*n* = 41) strongly and significantly associated with the presence of *STAT3* mutations (37 out of 41, 90.2%; χ^2^ = 49.5, *P <* 0.0001). Moreover, this immunophenotypic signature identified even almost all neutropenic patients (37 out of 41, 90.2%; χ^2^ = 49.5, *P* < 0.0001; Figure 3A), also including those 4 cases CD8+ T-LGL leukemia with neutropenia that had wild type *STAT3*. In more detail, among the 41 patients with CD16+/CD56- immunophenotype, 33 were both mutated and neutropenic, 4 were *STAT3* mutated without neutropenia and other 4 were neutropenic with wild type *STAT3*.

Within the 3 other immunophenotypes of CD8+ T-LGL leukemia any other case did not show neutropenia but one (1/27, 3.7%; Figure 3A), that was the only *STAT3* mutated case out of the most common immunophenotypic signature (CD16+/CD56-) and it belonged to CD16-/CD56-/CD57+ subset.

All the data are listed in Table 2. As indicated, CD16+/CD56- subgroup showed the ANC value significantly lower than that observed in the remaining CD8+ groups and CD4+ T-LGL leukemia patients (mean ANC ± SEM: 795.46 ± 80.03 mm^3^, 2,855.11 ± 224.54 mm^3^; 2,635.76 ± 193.81 mm^3^, respectively, *P* < 0.05; Figure 3B).

**Table 2:** Comparison of biological and clinical variables within CD8+ T-LGL leukemia between CD16+/CD56- and the other immunophenotypes

Variable	CD8+ T-LGL leukemia patients *n* = 68	CD16+/CD56- patients *n* = 41	Patients with other immunophenotypes *n* = 27	*P*
**Gender**				0.033
Males	**32 (47%)**	15 (37%)	17 (63%)	
Females	**36 (53%)**	26 (63%)	10 (37%)	
***STAT3* mutation**	**38 (56%)**	37 (90%)	1 (4%)	0.0001
**Neutropenia (ANC < 1,500)**	**38 (56%)**	37 (90%)	1 (4%)	0.0001
**Severe neutropenia (ANC < 500)**	**17 (25%)**	17 (41%)	0	0.0001
**Rheumatoid arthritis**	**3 (4%)**	3 (7%)	0	0.151
**Other autoimmune diseases**	**20 (29%)**	16 (39%)	4 (15%)	0.032
**Associated neoplasia**	**16 (24%)**	8 (20%)	8 (30%)	0.336
**Treatment**	**13 (19%)**	12 (29%)	1 (4%)	0.009

ANC, absolute neutrophil count.

Vbeta analysis was performed on the entire cohort of patients and no specific association was found between the presence of *STAT3* mutations, neutropenia and Vbeta usage. On the contrary, among CD4+ LGL leukemia patients a high frequency of Vbeta 13.1 was shown (9/29, 31%) as previously reported [6].

### Validation cohort

To confirm the correlation among immunophenotype, presence of *STAT3* mutations and neutropenia observed in the pilot study group, we evaluated an independent cohort of 20 patients from University Hospital Hematology Unit of Rennes, France. Specifically, we took into account a group of patients affected by CD8+ T-LGL leukemia and characterized by the immunophenotype of interest, CD16+/CD56- (Table 3). All samples were evaluated at diagnosis. In this cohort, 17 out of 20 patients were *STAT3* mutated and neutropenic. The remaining 3 patients were wild type for *STAT3* and only one was neutropenic. In this different cohort of patients, the presence of *STAT3* mutations and neutropenia resulted with an incidence of 85% and 90%, respectively, confirming the findings of the pilot cohort.

**Table 3:** Comparison of biological and clinical variables of the Italian and French cohorts of CD16+/CD56- CD8+ T-LGL leukemia patients

Variable	CD16+/CD56- CD8+ T-LGL leukemia patients (Padua, Italy) *n* = 41	CD16+/CD56- CD8+ T-LGL leukemia patients (Rennes, France) *n* = 20
**Gender**		
Males	15 (37%)	7 (35%)
Females	26 (63%)	13 (65%)
***STAT3* mutation**	37 (90%)	17 (85%)
**Neutropenia (ANC < 1,500)**	37 (90%)	18 (90%)
**Severe neutropenia (ANC < 500)**	17 (41%)	2 (10%)

ANC, absolute neutrophil count.

### STAT3 phosphorylation

**Figure 4:**
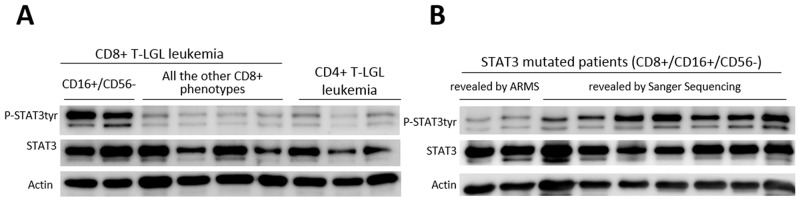
Western blot analysis of LGLs’ extracts for phosphorylated STAT3, total STAT3 and Actin **(A)** Representative cases for the patients subdivided according to their different immunophenotypes are reported. **(B)** Representative cases for *STAT3* mutated patients subdivided into samples where mutation was revealed only by ARMS-PCR (mutated clone < 25% of the entire clone) and those with mutations revealed by Sanger Sequencing. Actin expression is shown as gel loading control.

By western blot analysis, we showed that high STAT3 tyrosine phosphorylation was observed in LGL samples obtained by CD8+ T-LGL leukemia patients belonging to CD16+/CD56- subgroup, either they were mutated or wild type, either neutropenic or not. The average values, evaluated by densitometry on 26 samples, resulted statistically higher in these patients as compared with all the other immunophenotypic groups, evaluated on 23 samples (P-STAT3/STAT3, median ± SEM: 1.89 ± 0.78 and 0.49 ± 0.10, respectively, *P* < 0.01; Figure 4A). Interestingly, we observed that STAT3 phosphorylation was still present but lower in those patients whose mutation was detected only by ARMS-PCR, suggesting that STAT3 activation levels were likely correlated to the dimension of clone interested by mutation (Figure 4B).

### Fas ligand expression

**Figure 5:**
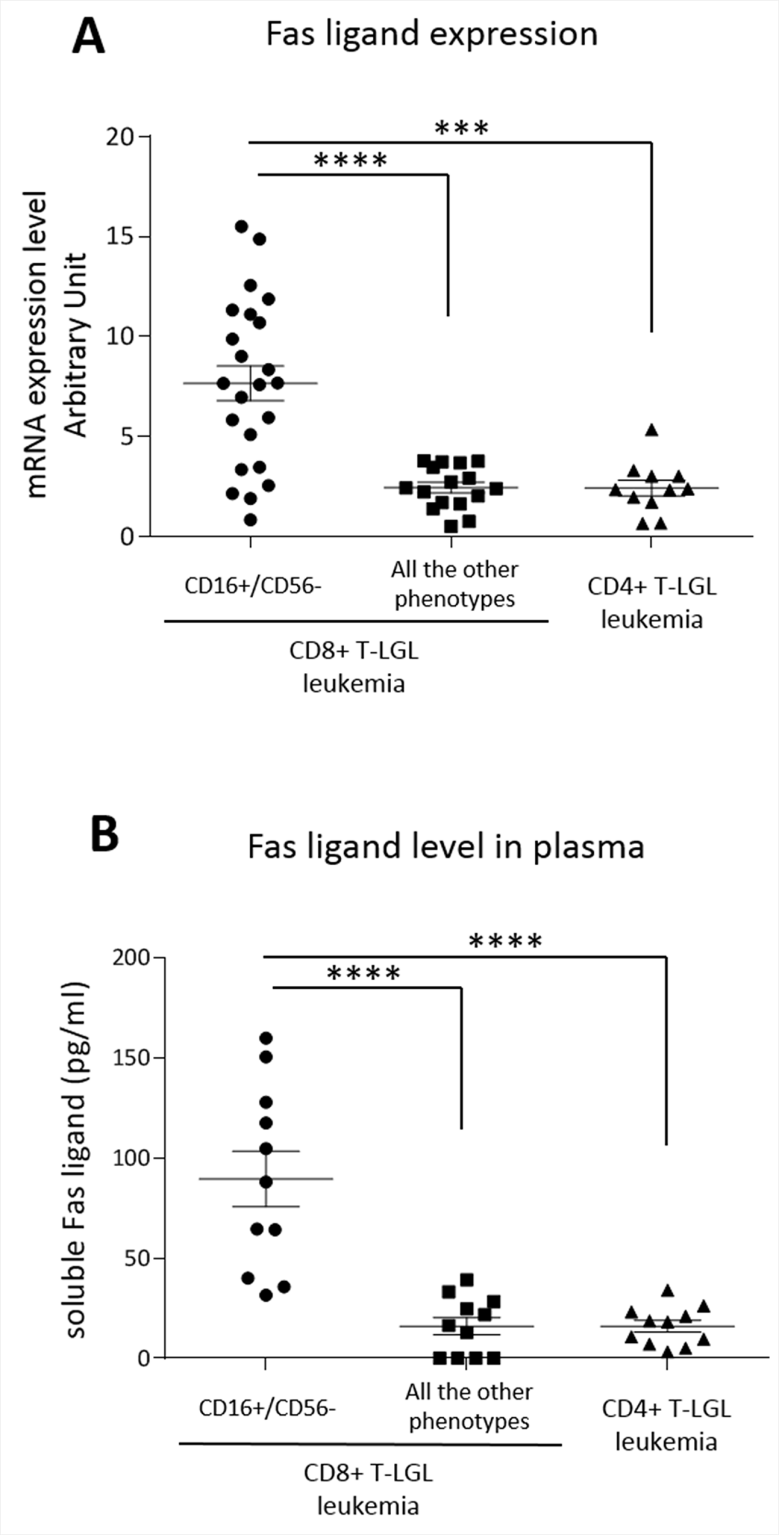
Fas ligand expression Dot plots report **(A)** mRNA transcription levels and **(B)** plasma levels of secreted Fas ligand of T-LGL leukemia patients subdivided according to their immunophenotypes, CD8+ T-LGL leukemia (distinguished in patients with CD16+/CD56- phenotype and those with all the other phenotypes) and CD4+ T-LGL leukemia. The means and SEM are reported. The expression level observed in the group of CD8+ T-LGL leukemia CD16+/CD56- subset is higher as compared with the two other groups (***P < 0.001, ****P < 0.0001, using one-way Anova and Tukey’s multiple comparison test).

Since secreted Fas ligand, which is increased in the serum of patients affected by T-LGL leukemia [20–22], has been hypothesized to play a role in inducing neutropenia in these patients [16], we evaluated Fas ligand expression in our series of patients subdivided according to their immunophenotype. By Real-Time PCR, we observed that Fas ligand transcriptional expression median level was higher in CD16+/CD56- CD8+ T-LGL leukemia patients as compared with the non-neutropenic patients belonging to the other immunophenotypes, both CD8+ T-LGL leukemia and CD4+ T-LGL leukemia (7.66 ± 0.87, 2.45 ± 0.22 and 2.35 ± 0.28 arbitrary units, respectively; *P* < 0.001; Figure 5A). The difference of Fas ligand production observed on transcriptional expression was also confirmed by ELISA measuring Fas ligand level in patients’ plasma (CD16+/CD56- CD8+ T-LGL leukemia: 88.3 ± 14.18 pg/ml, the other CD8+ and CD4+ immunophenotypes: 16.08 ± 14.62 pg/ml, *P* < 0.0001; Figure 5B).

### Fas ligand regulation

**Figure 6:**
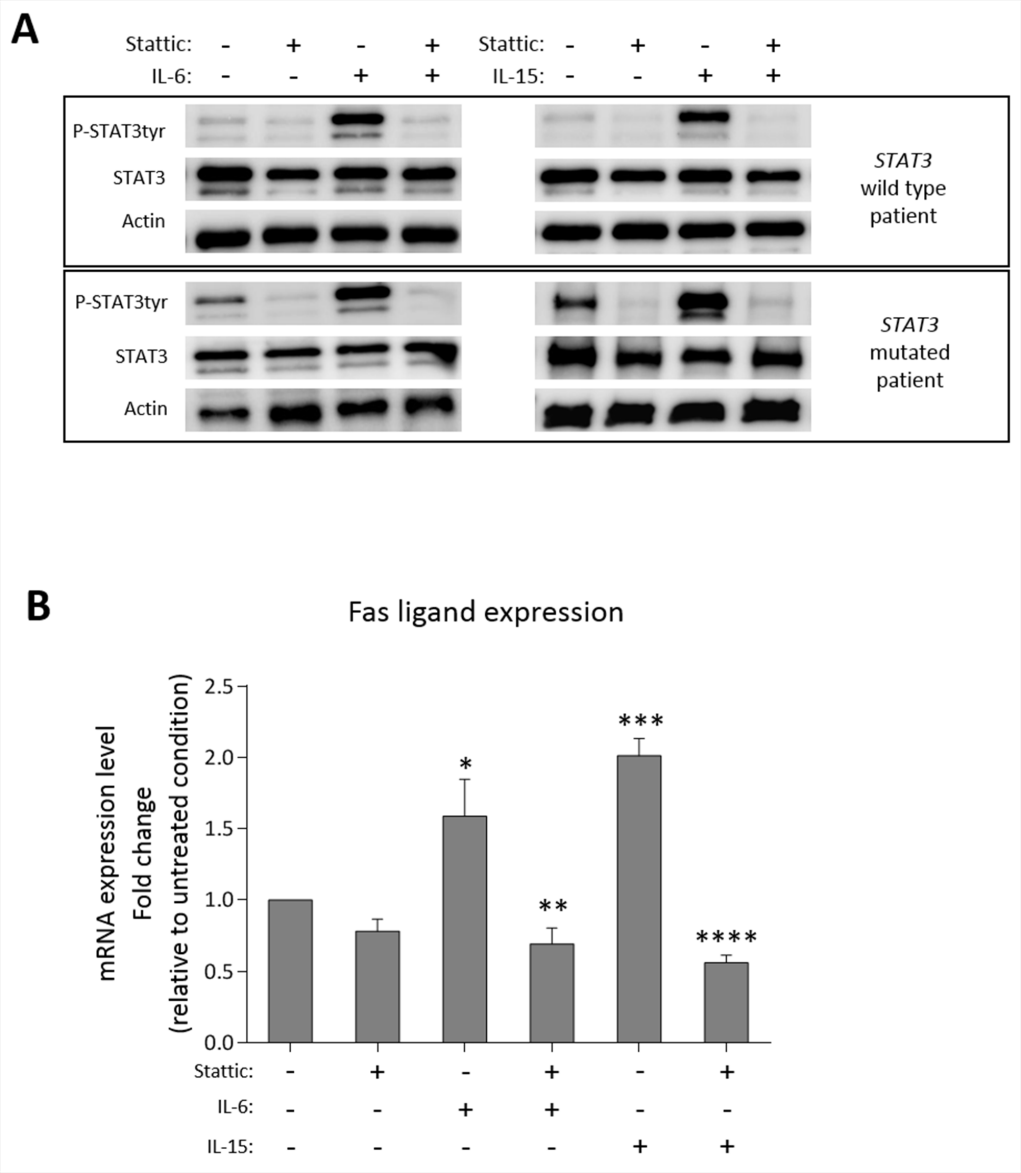
Fas ligand modulation after STAT3 activation/inhibition Western blot analysis and Real-Time PCR results of patients’ PBMCs after culture in the following different conditions: untreated condition (UT) for 2 hours; with Stattic (15 μM) for 2 hours; stimulated by IL-6 (20 ng/ml) or IL-15 (20 ng/ml) for 1 hour; pretreated for 1 hour with Stattic and then stimulated by IL-6 or IL-15 for 1 hour. **(A)** STAT3 expression and tyrosine phosphorylation in whole-cell extracts were analyzed. Actin expression is shown as gel loading control. Upper panels show a representative *STAT3* wild type sample, lower panels a representative *STAT3* mutated sample. Data from two representatives out of six independent experiments are shown. **(B)** The histogram reports the average fold change of Fas ligand mRNA of patients’ PBMCs. All the values were settled on untreated condition set at 1.0. Data are represented as mean ± SEM of six independent experiments. *P < 0.05 vs. UT; **P < 0.01 vs. IL-6 condition; ***P < 0.001 vs. UT; ****P < 0.0001 vs. IL-15 condition, using one-way Anova and Tukey’s multiple comparison test.

To evaluate whether the high level of Fas ligand transcription depends on STAT3 activation, we treated PBMCs of patients in culture with Stattic, the specific inhibitor of STAT3 activation [23]. We observed that both STAT3 phosphorylation (Western Blot analysis, Figure 6A, wild type *STAT3* on the upper panel and mutated *STAT3* on the lower panel) and Fas ligand transcription (Real-Time PCR analysis, Figure 6B) slightly decreased after 2 hours culture with Stattic (15 μM) as compared to the untreated conditions. Consistently, when we triggered STAT3 phosphorylation (Figure 6A) with IL-6 (20 ng/ml) or IL-15 (20 ng/ml), key cytokines in LGL leukemia development [9, 24], after one hour culture we observed an increase of Fas ligand transcription levels (1.59- and 2.01-fold after IL-6 and IL-15, respectively, Figure 6B). To verify whether Fas ligand expression was modulated just by STAT3 activation, before ILs incubation we pre-treated patients’ PBMC for 1 hour with Stattic. We observed that Stattic blocked IL-6 or IL-15 effects preventing STAT3 phosphorylation induction (Figure 6A) and the increase of Fas ligand transcription (Figure 6B), thus demonstrating that the induction of Fas ligand expression is mediated by P-STAT3. This mechanism was observed in each immunophenotypic category of LGL leukemia patients, in detail, both STAT3 mutated patients and wild type patients, presenting different P-STAT3 and Fas ligand levels, showed similar molecular responses to IL-6 or IL-15 activation and Stattic inhibition. Our data demonstrated that STAT3 activation is crucial in Fas ligand regulation and explained the high levels of Fas ligand in CD8+/CD16+/CD56- patients characterized by high STAT3 activation.

### *STAT5b* mutations

According to a recent report [13] showing high incidence of *STAT5b* mutations in CD4+ T-LGL leukemia, we evaluated also the presence of *STAT5b* mutations in all our cohort of study.

In our cohort, *STAT5b* mutations, represented by N642H (*n* = 2), Y665F (*n* = 2) and Q706L (*n* = 1), were found in 5 out of 101 patients (5%). Interestingly, *STAT5b* mutations were present only in CD4+ T-LGL leukemia patients, confirming the data reported by Andersson et al [13], although with a lower incidence of 15.2% in CD4+ T-LGL leukemia (5 out of 33 patients) compared to 55% frequency reported by others, likely because of the different number of patients studied (33 versus 11). Consistent with all CD4+ T-LGL leukemia patients, *STAT5b* mutated cases did not show any symptomatic clinical manifestation.

At variance to *STAT3* mutated patient, analyzing CD16, CD56, CD57 cell markers no specific immunophenotype distinguishing *STAT5b* mutated from wild type patients was evidenced among CD4+ T-LGL leukemia. In fact, all CD4+ patients showed only two immunophenotypes, 78.8% characterized by the CD16-/CD56+/CD57+ pattern and 21.2% characterized by the CD16+/CD56+/CD57+ pattern. *STAT5b* mutated patients were all equipped with the most frequent CD16-/CD56+/CD57+ immunophenotype. Vbeta 13.1 prevalence observed in CD4+ T-LGL leukemia was similarly found in *STAT5b* mutated patients (2/5, 40%).

## DISCUSSION

In a large cohort of T-LGL leukemia patients we showed that the CD8+/CD16+/CD56- LGL phenotype correlates with a specific subset of T-LGL leukemia patients, characterized by *STAT3* mutations and neutropenia. To get insights into the molecular bases of neutropenia in these patients, we demonstrated a link between STAT3 phosphorylation and Fas ligand transcription levels. Supporting the correlation between mutational pattern and leukemic LGL phenotype, *STAT5b* mutations were specifically found only in CD4+ T-LGL leukemia.

At present *STAT* mutations are the most distinctive genetic lesions described in this disease and represent a molecular marker with high specificity [25], although not exclusive [26–28] of this lymphoproliferative disorder. At variance with other hematological conditions (i.e., Hairy Cell Leukemia, Waldenstrom Macroglobulinemia) [29, 30], the clinical significance of *STAT* mutations in patients with T-LGL leukemia has not been yet clarified. Some literature data correlated the presence of *STAT3* mutations with neutropenia [10, 14], but these data were not confirmed by other authors [11, 31], moreover this correlation was not yet specifically evaluated in comparable series of cases. At variance *STAT5b* mutations have been reported to have a different clinical impact depending on which subset they were found, having been described to be associated to CD4+ T-LGL leukemia, characterized by indolent disease, [13] but having also been found in two cases of the rare aggressive form of LGL leukemia disorder (one with NK and the other with T type) [12].

Showing a close relationship between *STAT3* genetic lesion and the presence of neutropenia, our results contribute to clarify the link between *STAT3* mutations and symptomatic disease and, for the first time in a large series of patients, demonstrate an association between a specific immunophenotype and discrete biological and clinical features. Moreover, our results provide further evidence of the correlation between *STAT5b* mutations and CD4+ T-LGL leukemia, recently described in a small cohort of cases (*n* = 11) and here confirmed in a larger cohort of patients (*n* = 33) even if with a lower incidence (15.2% vs 55% reported [13]).

Interestingly, our results indicate that CD8+ and CD4+ T-LGL leukemia present different biologic background. In fact, *STAT3* and *STAT5b* mutations represent an exclusive marker of CD8+ T-LGL leukemia and CD4+ T-LGL leukemia, respectively. Among the different CD8+ T-LGL leukemia immunophenotypic combinations, patients characterized by CD16+/CD56- proliferating cells showed the highest frequency of *STAT3* mutations. Only 4 out of 41 cases within this specific immunophenotypic subgroup were devoid of *STAT3* mutations. It might be speculated that *STAT3* mutations could indeed occur in these patients, but present in only a very low subset of LGL clone (ARMS is currently available only for the two main mutations, Y640F and D661Y) or likely located at sites not yet screened. Supporting this last hypothesis, in our study group we identified also mutations that are not already reported, located on exon 21, where the main mutations have been found, more precisely in-frame deletions/insertions (Figure 1). Furthermore, a recent paper described mutations that, even if located outside the SH2 domain, were able to induce a gain of function of STAT3 [32]. On this basis we can speculate that mutation analysis might actually underestimate the real incidence of *STAT3* genetic lesions. To strength the significance of our results, they were validated on a cohort of 20 patients coming from France, confirming that the CD8+/CD16+/CD56- immunophenotype might have a prognostic value in T-LGL leukemia patients, thus contributing to the identification of patients most at risk of neutropenia, for whom *STAT3* mutation analysis is highly recommended. This validation, based on retrospective data, takes into account only patients with the phenotype of interest within the French cohort. A larger validation cohort including all the possible phenotypes would have been preferred, but it would have been possible only by performing a multicentre study unifying the criteria of evaluation of all the different variables. Concerning this point, according to our experience, we emphasize that a correct immunophenotype characterization must be performed not only in whole blood but also on purified PBMCs, since total blood CD16 population is frequently underestimated or even unscreened because of the anti-CD16 specific antibody binding to granulocytes.

In terms of the mechanisms accounting for neutropenia in CD8+/CD16+/CD56- phenotypic subset characterized by *STAT3* mutations, our data suggest a pathogenetic link between STAT3 activation and the development of neutropenia. According to the finding that mutations confer STAT3 activation [10], we observed higher P-STAT3 in mutated patients. Interestingly, high levels of P-STAT3 tyr705 were demonstrated mostly on CD8+/CD16+/CD56- LGL patients and also in the 4 patients of this subset lacking *STAT3* mutations, suggesting that a strong STAT3 activation might represent a specific hallmark of this immunophenotypic subset. Consistently, STAT3 activation in other immunophenotypic subsets was lower. Liu and co-workers [16] showed that neutropenia in LGL leukemia patients was consequent to the high level of circulating Fas ligand produced by LGLs, since neutrophils undergo apoptosis through Fas triggering. Here we provide evidence that Fas ligand expression was specifically higher in patients characterized by CD8+/CD16+/CD56- phenotype as compared to other subsets. We also demonstrated that this higher Fas ligand transcription was mediated by STAT3 activation. In fact, we found that Stattic, a specific inhibitor of STAT3 phosphorylation, was able to reduce Fas ligand transcription levels. Furthermore, inducing STAT3 phosphorylation with IL-6 or IL-15 we observed an increase of Fas ligand transcription that was prevented through Stattic exposure. These results might explain why Fas ligand was found to be more expressed in CD8+/CD16+/CD56- patients, who are specifically characterized by higher level of P-STAT3.

Confirming the well-known heterogeneity of T-LGL leukemia, this study, performed in a large cohort of patients, provides evidence for a link between biological markers (phenotype, *STAT3* mutations and activation, Fas ligand production) and neutropenia. Our results also contribute to clearly separate CD4+ from CD8+ T-LGL leukemia, the first group being characterized by very low frequency of neutropenia and lack of *STAT3* mutations.

In conclusion, our data emphasize the relevance of flow cytometry and *STAT3* mutation analysis in order to gain information on clinical course and biologic features of disease, thus correctly addressing the management of each patient. Moreover, our results show that already at diagnosis flow cytometry evaluation might represent a valuable tool to identify patients who need further investigation.

## MATERIALS AND METHODS

### Study patients

The pilot study population consisted of 101 patients affected by T-LGL leukemia from Hematology Unit of Padua (Italy). Additional 20 patients, representing a validation cohort, were recruited from Hematology Unit of Rennes (France). All 121 patients met the 2008 WHO (World Health Organization) criteria for T-LGL leukemia diagnosis with LGL expansion (>500/μl) persisting for at least 6 months; clonality was demonstrated by molecular analysis of T-cell receptor (TCR) gene rearrangement in all cases. Patients characteristics were evaluated, including the presence of cytopenia, association with autoimmune disease, secondary neoplasia and treatment requirement. Median follow-up was 9 years, ranging from 3 to 16 years. Neutropenia, particularly severe neutropenia, was confirmed in repeated ANC performed every 1-6 months depending on individual clinical characteristics during the follow-up.

All samples were recruited at diagnosis and during the disease follow-up. This study was performed according to the Helsinki Declaration and patients gave written informed consensus prior to inclusion in the study. The study and blood sample collection were approved by Ethic Committee for Clinical Trial of Padua.

### Flow cytometry analysis

The frequency of LGLs positive for the charac-teristic antigens was assessed by flow cytometry analysis using direct or indirect immunofluorescence assay combining up to 6 fluorescences. Briefly, cells were stained with the appropriate mAbs either unlabeled or labeled with different fluorochromes; staining with unlabeled mAb was followed by conjugated isotype-specific goat anti-mouse secondary reagent (Southern Biotechnology, Birmingham, AL, or Caltag, Burlingame, CA). The commercially available FITC-, phycoerythrin (PE)-, PeCy5-conjugated, PeCy7-conjugated, APC-conjugated and APC-Cy7-conjugated mouse monoclonal antibodies (mAbs) used included: anti-CD3 (SK7), anti-CD4 (RPA-T4), anti-CD8 (RPA-T8), anti-CD16 (3G8), anti-CD56 (NCAM16.2) and anti-CD57 (NK-1) from Becton Dickinson (Sunnyvale, CA, USA).

Cells were scored using a FACSCanto analyzer (BD Biosciences, San Jose CA) and data processed by the BD FACSDiva software program (BD Biosciences). The investigation for LGL surface markers (CD3, CD4, CD8, CD16, CD56, CD57) was performed on peripheral blood and repeated also on fresh PBMCs, since in total blood CD16 was frequently underestimated or even unscreened because the specific antibody anti-CD16 was sequestered by granulocytes.

### Isolation of LGLs from patients with T-LGL leukemia

PBMCs were obtained by Ficoll-Hypaque (Sigma Aldrich) gradient centrifugation. Patients’ T-LGLs were obtained using magnetic separations over columns (MACS; Miltenyi Biotec, Auburn, CA) with magnetic Micro-Beads coated with monoclonal anti-human CD57, CD56 or CD16 antibodies (Miltenyi Biotec). Alternatively, LGLs were obtained from PBMCs by the FACSAria cell sorter (BD Biosciences) making the selection according to CD57, CD56 or CD16 antigen expression. Sorted populations were analyzed for purity and viability (both > 95%). In preliminary experiments we ruled out that cells obtained by FACSAria sorting or by magnetic MicroBeads were functionally different.

### Screening of STAT mutations

For the screening of *STAT3* and *STAT5b* mutations we used the set of primers reported by Koskela et al [10] and by Rajala et al [12], respectively, to amplify the hot spot regions for mutations (exon 21 for *STAT3* and exons 16-18 for *STAT5b*). DNA from purified LGLs and from the remaining autologous PBMCs was separately analyzed. DNA was extracted from 1-20 × 10^6^ cells using the Puregene Cell Kit Plus (Qiagen) and then sequenced using dye terminator technology and an ABI 3130 sequencer (Applied Biosystems). The presence of D661Y and Y640F *STAT3* mutations undetectable by direct sequencing, because of the limited sensitivity of the method (reaching 25% of positive cells, as previously established) [9], was also analyzed by a DNA tetraprimer amplification refractory mutation system assay (ARMS-PCR), as reported by Jerez et al [11].

### Western blot analysis

Cells (2.5 × 10^5^ for each assay) were boiled for 7 minutes in Laemmli sample buffer. Samples were then subjected to sodium dodecyl sulfate/polyacrylamide gel electrophoresis (acrylamide 10% gels), transferred to PVDF membranes, immunostained with the specific antibodies and at the end with monoclonal anti-β-actin and revealed using an enhanced chemiluminescent detection system (Thermo Scientific; Waltham, MA, USA). The blots were acquired with the ImageQuant LAS 500 and analyzed by ImageQuant TL v8.1 software (GE Healthcare; Buckingamshire, UK). For Western blot analysis the following primary or secondary antibodies were used: polyclonal rabbit anti-STAT3 (79D7) and anti-phospho STAT3 Tyrosine 705 (D3A7) antibodies from Cell Signaling (Danvers, MA); monoclonal mouse anti-β-actin (AC-15) antibody from Sigma Aldrich (St. Louis, MO); horseradish peroxidise-conjugated anti-rabbit and anti-mouse immunoglobulins from PerkinElmer (Waltham, MA, USA).

### Real-Time PCR

Total cellular RNA was extracted from cells using RNeasy Mini Kit (Qiagen, Hilden, Germany), according to the manufacturer’s protocol and treated with DNase (Qiagen). Complementary DNA was generated from 1 μg total RNA using oligo-dT primer and the AMV reverse transcriptase (Promega, Madison, WI, USA). Real-Time polymerase chain reaction was carried out in an ABI Prism 7000 sequence detection system (Applied Biosystems, Foster City, CA). SYBR Green PCR Master Mix was purchased from Roche. The primers used are: Fas ligand, forward 5’-GCTGCCACCCCTGAAGAA-3’, reverse 5’-ATGAAAAACATCACAAGGAGACACA-3’; GAPDH (glyceraldehyde-3-phosphate dehydrogenase), forward 5’-AATGGAAATCCCATCACCATCT-3’, reverse 5’-CGCCCCACTTGATTTTGG-3’. The primers were designed by Primer Express 3.0 (Applied Biosystems). The results were obtained with Delta Delta Ct analysis. The relative amounts of mRNA were normalized for GAPDH expression.

### Cell culture

PBMCs from patients were cultured at 2×10^6^ cells/ml in RPMI-1640 (EuroClone) medium, supplemented with 10% fetal calf serum, 100 U of penicillin and 100 μg streptomycin (EuroClone) per ml and grown in 5% CO_2_ at 37°C. We left cell culture untreated for 24 hours, then we treated with Stattic (15 μM, Selleckchem) for one hour, specifically inhibiting STAT3 phosphorylation, dimerization and nuclear translocation [23], prior to stimulation by IL-6 (20 ng/ml; Sigma Aldrich) or IL-15 (20 ng/ml; Peprotech) for one hour.

### Enzyme-linked immunosorbent assay (ELISA)

Levels of secreted Fas ligand in plasma were determined by ELISA kit (RayBiotech, Georgia, USA), following the manufacturer’s recommendations. A standard curve was generated using seven solutions of recombinant human Fas ligand with known concentrations (1,000, 333.3, 111.1, 37.04, 12.35, 4.12, and 1.37 pg/ml). Briefly, 100 μl of each standard or sample, in duplicate, was added to each well of a 96-well ELISA plate and incubated 2.5 hours at room temperature. After four consecutive washes (4×300μl with Wash Buffer), 100 μl of biotin antibody was added to each well and incubated at room temperature, for 1 hour. After a second washing step (4×300 μl), 100 μl of Streptavidin solution was added and incubated at room temperature for 45 minutes. After a third washing step (4×300 μl), 100 μl of tetramethyl-benzidine (TMB) substrate reagent was added to each well and incubated for 30 minutes at room temperature in the dark. The reaction was interrupted by adding 50 μl of stop solution per well and absorbance was immediately read at 450 nm by a microplate reader platform (Victor Multilabel plate reader, PerkinElmer).

### Statistics

Data are expressed as mean plus or minus the standard error mean (SEM) and statistical analysis was performed by one-way Anova followed by Tukey’s multiple comparison test. Comparisons of proportions and ranks of variables between groups were performed by χ^2^ test. All the analyses were undertaken using GraphPad Prism 6. A value of *P* < 0.05 was accepted as significant.

## Abbreviations

LGL: large granular lymphocyte
KIR: killer immunoglobulin-like receptor
AICD: activation induced cell death
ARMS: amplification refractory mutation system
STAT3: signaling transducer and activator of transcription 3
SOCS3: suppressor of cytokine signaling 3
PBMC: peripheral blood mononuclear cells
ANC: absolute neutrophil count
RA: rheumatoid arthritis
MTX: methotrexate
WHO: World Health Organization
TCR: T-cell receptor
ELISA: Enzyme-linked immunosorbent assay
UT: untreated condition
